# Nitridation Temperature Effect on Carbon Vanadium Oxynitrides for a Symmetric Supercapacitor

**DOI:** 10.3390/nano9121762

**Published:** 2019-12-11

**Authors:** Ndeye M. Ndiaye, Ndeye F. Sylla, Balla D. Ngom, Bridget K. Mutuma, Julien K. Dangbegnon, Sekhar C. Ray, Ncholu Manyala

**Affiliations:** 1 Department of Physics, Institute of Applied Materials, SARChI Chair in Carbon Technology and Materials, University of Pretoria, Pretoria 0028, South Africa; nmaty.ndiaye@gmail.com (N.M.N.); ntoufasylla@gmail.com (N.F.S.); bridgetmutuma@gmail.com (B.K.M.); dangbegnon01@googlemail.com (J.K.D.); 2 Laboratoire de Photonique Quantique d’Energie et de NanoFabrication, Groupe de Physique du Solide et Science des Materiaux, Departement de Physique FST-UCAD BP 5005 Dakar-Fan, Dakar 999066, Senegal; bdngom@gmail.com; 3 Department of Physics, College of Science, Engineering and Technology, University of South Africa, Private Bag X6, Florida 1710, Science Campus, Christiaan de Wet and Pioneer Avenue, Florida Park, Johannesburg 1710, South Africa; raysc@unisa.ac.za

**Keywords:** carbon-vanadium oxynitride, mesoporous, pore size distribution, nitridation temperature, symmetric capacitor

## Abstract

In this work, porous carbon-vanadium oxynitride (C-V_2_NO) nanostructures were obtained at different nitridation temperature of 700, 800 and 900 °C using a thermal decomposition process. The X-ray diffraction (XRD) pattern of all the nanomaterials showed a C-V_2_NO single-phase cubic structure. The C-V_2_NO obtained at 700 °C had a low surface area (91.6 m^2^ g^−1^), a moderate degree of graphitization, and a broader pore size distribution. The C-V_2_NO obtained at 800 °C displayed an interconnected network with higher surface area (121.6 m^2^ g^−1^) and a narrower pore size distribution. In contrast, at 900 °C, the C-V_2_NO displayed a disintegrated network and a decrease in the surface area (113 m^2^ g^−1^). All the synthesized C-V_2_NO yielded mesoporous oxynitride nanostructures which were evaluated in three-electrode configuration using 6 M KOH aqueous electrolyte as a function of temperature. The C-V_2_NO@800 °C electrode gave the highest electrochemical performance as compared to its counterparts due to its superior properties. These results indicate that the nitridation temperature not only influences the morphology, structure and surface area of the C-V_2_NO but also their electrochemical performance. Additionally, a symmetric device fabricated from the C-V_2_NO@800 °C displayed specific energy and power of 38 W h kg^−1^ and 764 W kg^−1^, respectively, at 1 A g^−1^ in a wide operating voltage of 1.8 V. In terms of stability, it achieved 84.7% as capacity retention up to 10,000 cycles which was confirmed through the floating/aging measurement for up to 100 h at 10 A g^−1^. This symmetric capacitor is promising for practical applications due to the rapid and easy preparation of the carbon-vanadium oxynitride materials.

## 1. Introduction

The growing demand for conversion of renewable energy systems and a foreseeable shortage of fossil fuels for usable energy is a major challenge for energy storage. However, many high-energy storage systems have also been developed to meet the generated energy efficiently for productive use and cost-effectiveness, such as batteries and supercapacitors [1,2]. Supercapacitors (SCs), also named ultracapacitors, have largely been studied in the field of energy storage. They exhibit a high power density, a longer cycling life, and safety as compared to batteries [3,4]. However, ultracapacitors possess low energy [5], and their improvement may be achieved by the synthesis of materials that display unique morphologies with high performance.

Typically, ultracapacitors can be classified into two types based on their charge storage mechanism: electric double-layer capacitors (EDLCs) and Faradaic capacitors [6,7]. EDLCs can store the charge in double-layer capacitance. The materials used are carbon-based active materials with high specific surface area (SSA), good electronic conductivity, good stability and low specific capacitance [6]. In pseudocapacitive or Faradaic capacitors, the charge storage mechanism is based on fast reversible redox reactions such as in metal oxides (MOs), metal hydroxides (MOHs) and conducting polymers (CPs) [8,9].

For decades, metal oxides have been widely used for energy storage applications due to their high Faradaic activity and their variable oxidation states by adopting different structures [10]. Nevertheless, metal oxides suffer low SSA and low conductivity which leads to low specific power and low cyclic stability [11]. Thus, it is necessary to synthesize new electrode materials applicable in Faradaic capacitors. Recently, several achievements geared at improving the stability, conductivity and wettability of Faradaic materials have been reported such as the adoption of metal sulphides (MSs) [12], metal nitrides (MNs) [13] and metal oxynitride (MONs) [14]. For MNs, the oxygen (O^2−^) atoms in the metal octahedral position were completely replaced by nitrogen (N^3−^) atoms, while for the MONs (e.g., M = Ti [15], W [16], Mo [17], V [18]), the replacement is partial which leads to the presence of some metal oxides [14].

Among the greatest recent achievements, vanadium oxynitride materials has been considered to be an excellent candidate due to their superior outstanding electrochemical properties due to multiple oxidation states (V^5+^, V^4+^, V^3+^, V^2+^) leading to an excellent specific capacity, and the presence of some vanadium oxides. VO_x_N_y_ still suffers from poor rate performance, low specific surface area and aging effects [14,19]. However, most reports on vanadium oxynitride materials entail the use of vanadium oxide precursors such as VO_2_ or V_2_O_5_ resulting in the generation of aggregated VO_x_N_y_ particles owing to the low melting point of the vanadium oxides [20]. Consequently, this limits the electrochemical performance of these materials due to their low specific surface areas [18,20]. To overcome this drawback, an appropriate and efficient vanadium precursor is required.

Although various researchers have reported on the use of vanadium chloride and vanadium oxychloride precursors to obtain good crystalline VO_x_N_y_ nanostructures [18,21], a controlled working environment is required due to the high reactivity and toxicity of the chlorides [22]. Ammonium metavanadate (NH_4_VO_3_) is less toxic, less corrosive and safer compared to the chloride-based precursors and thus, a suitable alternative for the preparation of VO_x_N_y_ materials. Moreover, a recent approach on the use of organic precursors in the growth of oxynitride materials has resulted in a controlled particle formation, generation of a crystalline structure, as well as the ability to tune the specific surface area of the final product [23]. In this regard, polyvinylpyrrolidone, urea, cyanamide and melamine are commonly used organic precursors as they serve as both carbon and nitrogen sources with the melamine providing a higher nitrogen and carbon contents. For instance, Cheng et al. [20] studied VN_1.02_O_0.1_ material by calcining a mixture of melamine and V_2_O_5_ xerogel in a furnace at 800 °C under N_2_ atmosphere. They obtained a carbon content of 52.6% and a nitrogen content of 22.6% after melamine decomposition [20]. The presence of carbon in the vanadium oxynitride matrix can greatly influence the specific surface area, structure and morphology of the VO_x_N_y_ and, as a result, improve their electrochemical capacitance [22]. Recently, a different approach of tuning nitridation temperature of oxynitride based-materials has been proposed to enhance their properties. For example, Jacke et al. [24] demonstrated the dependence of the conductivity of lithium-phosphorous oxynitride films on temperature (at room temperature, 200 °C and 300 °C). They concluded that an increased amount of nitrogen revealed an improvement in the conductivity of the oxynitride materials at high temperatures [24]. To the best of our knowledge, to date no reports on the evaluation of vanadium oxynitride materials at different nitridation temperature for supercapacitors have been found in the literature.

This study presents an in-depth understanding of how nitridation temperature influences the physical and electrochemical properties of C-V_2_NO nanostructures. At 700 °C, a low surface area and less crystalline C-V_2_NO nanostructure was obtained. In contrast, at 800 and 900 °C, the C-V_2_NO nanostructures showed higher specific surface area and smaller pore size distribution. The effect of the different nitridation temperature was associated with the electrochemical performances of the C-V_2_NO electrodes evaluated in 6 M KOH aqueous electrolyte. To confirm the electrochemical properties of the material, a symmetric supercapacitor (C-V_2_NO//C-V_2_NO) was assembled using a C-V_2_NO@800 °C in both positive and negative potential windows. In a wide voltage of 1.8 V, the symmetric device exhibited a specific energy and specific power of 38 W h kg^−1^ and 764 W kg^−1^ at 1 A g^−1^, respectively. The (C-V_2_NO//C-V_2_NO) showed 84.7% capacity retention at 10,000th cycles and excellent voltage holding stability up to 100 h at a high specific current.

## 2. Experimental

### 2.1. Synthesis of C-V_2_NO Nanomaterials by Thermal Decomposition Technique

**Scheme 1:**
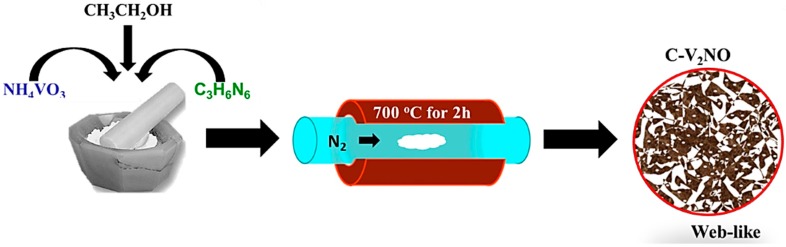
**:** Preparation steps of carbon-vanadium oxynitride at 700 °C.

Scheme 1 presents the diagram illustrating the procedure adopted to synthesize the C-V_2_NO nanomaterials at a nitridation temperature of 700 °C. The C-V_2_NO nanomaterials were synthesized by thermal decomposition method using ammonium metavanadate (NH_4_VO_3_, 99%) and melamine (C_3_H_6_N_6_, 99%) with a mass loading ratio of 1:10. The preparation details of the C-V_2_NO was reported in our previous study by Ndiaye et al. [25]. Briefly the NH_4_VO_3_ and C_3_H_6_N_6_ powders were mixed in an agate mortar with a few drops of ethanol (CH_3_CH_2_OH, 99%) to make a homogeneous slurry. This was inserted into a quartz tube furnace and heated to 700 °C at a heating rate of 18 °C min^−1^ before being kept at that temperature for 2 h under a stream of nitrogen (N_2_) gas flow. After cooling down to room temperature, the final product was obtained and labelled as C-V_2_NO@700 °C. The same method has been used to prepare C-V_2_NO nanomaterials at 800 °C and 900 °C.

### 2.2. Structure, Morphology and Composition Characterization

X-ray diffraction (XRD) analysis of as-prepared C-V_2_NO nanomaterials was studied using a cobalt X-ray source at 50 mA and 35 kV by a XPERT-PRO diffractometer (PANalytical BV, Amsterdam, Netherlands) with θ/2θ geometry in the range from 10–90°. Raman spectroscopy analysis of the materials were operated using a WITec confocal Raman microscope (WITec alpha300 RAS+) (WITec, Ulm, Germany). A field-emission scanning electron microscope of a Zeiss Ultra plus 55 field (FE-SEM) (Akishima, Japan) was used to investigate the morphology of the materials and the elemental mapping operated at a voltage of 2.0 kV. Transmission electron microscopy (TEM) analysis was carried out at 200 kV on a JEOL JEM-2100F microscope (Akishima-shi, Japan).

X-ray photoelectron spectroscopy (XPS, Thermo Fisher, Waltha, MA, USA, K-alpha) was used to study the composition of the materials with a monochromatic Al-Kα radiation. The porosimetry tests of the C-V_2_NO nanomaterials were evaluated with nitrogen adsorption-desorption isotherms at −196 °C using a Micromeritics ASAP 2020 (Tristar, Norcross, GA, USA). The specific surface area values of the materials were obtained by the Brunauer–Emmett–Teller (BET) analysis using the adsorption branch in the relative pressure range (P/P0) of 0.01–1.0.

### 2.3. Electrochemical Characterization of C-V_2_NO Electrodes

A Bio-Logic VMP-300 (Knoxville TN, USA) potentiostat were used to evaluate the electrochemical measurements (three- and two-electrode configurations) of the C-V_2_NO electrodes at room temperature. The C-V_2_NO electrodes were investigated by mixing weighed amounts of C-V_2_NO active material, carbon black additive and polyvinylidene difluoride (PVDF) binder in a mass ratio of 80:10:10 with *N*-methylpyrrolidone (NMP) as a solvent. The slurry was applied on a clean nickel foam (NiF) with a 3D scaffold template as a current collector. The electrodes were kept at 60 °C in an electric oven overnight to ensure a complete evaporate of the NMP. Ag/AgCl (3 M KCl saturated) was used as reference electrode, a glassy carbon plate as counter electrode in a 6 M KOH aqueous electrolyte. The C-V_2_NO electrodes were used as working electrodes to evaluate the electrochemical performance.

The measurements of the cyclic voltammetry (CV) and galvanostatic charge-discharge (GCD) were studied to determine the behaviour, the stable working potential and to calculate the specific capacity and the stability test of the electrodes, respectively. The specific capacity (Q_S_, mA h g^−1^) of the electrodes’ materials was calculated using Equation (1) [26].

(1) $$Q=\frac{I_{d\;}\times t_{D}}{3.6}$$

where *I_d_* is defined as the specific current measured in A g^−1^ and *t_D_* is the time in second (s) for a complete discharge cycle.

The specific energy (*E_d_*,W h kg^−1^) and the specific power, (*P_d_*, W kg^−1^) of the C-V_2_NO//C-V_2_NO device were calculated using Equations (2) and (3) at different specific currents [27].

(2) $$E_{d}\left(W\;h\;kg^{-1}\right)=\frac{I_{d}}{3.6}\int^{\;}V\left(t\right)dt$$

(3) $$P_{d}\left(W\;kg^{-1}\right)=3600\frac{E_{D}}{t_{D}}$$

where *I_d_* (A g^−1^) represents the specific current, *t_D_* (s) is called discharge time from the GCD curve, and *V* (V) is the voltage of the symmetric capacitor.

The measurements of the electrochemical impedance were carried out to study the conductivity and capacitive properties of the electrodes from 100 kHz to 10 mHz using an open circuit voltage.

## 3. Results and Discussion

**Figure 1:**
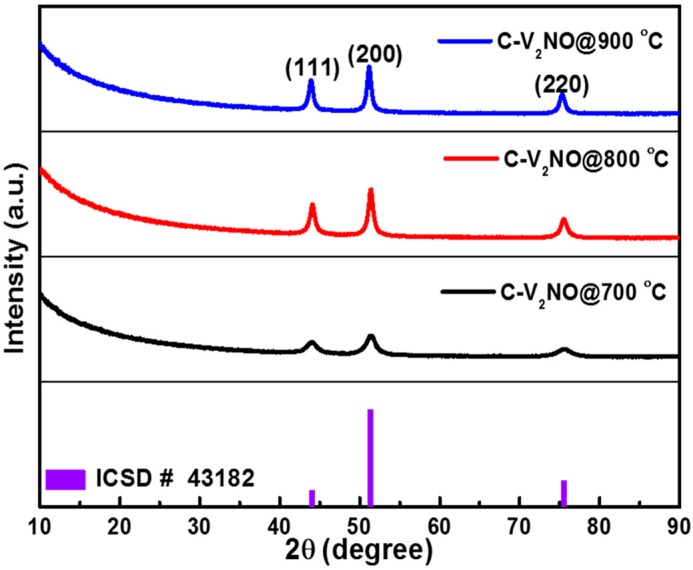
The X-ray diffraction (XRD) patterns of the C-V_2_NO materials grown at 700, 800 and 900 °C.

XRD analysis results of the carbon-vanadium oxynitride nanostructures synthesized at 700, 800 and 900 °C, respectively, are presented in Figure 1 The XRD patterns of the materials showed three diffraction peaks confirming the crystallinity of the material. The diffraction peaks are located at 2θ value of 44.0°, 51° and 75.5° corresponding to the (111), (200) and (220) crystallographic planes, respectively. The XRD pattern of the nanomaterials shows C-V_2_NO cubic nanostructure using the matching inorganic crystal structure Database (ICSD) card #43182 [25]. The peak intensity of the C-V_2_NO was found to increase with nitridation temperature indicating an increase in crystallinity. Indeed, the peaks of C-V_2_NO@800 °C and C-V_2_NO@900 °C were narrower and much pronounced than the ones for C-V_2_NO@700 °C. However, the diffraction peak of carbon was not observed in Figure 1. This is due to the amorphous nature of the carbon in the materials.

**Figure 2:**
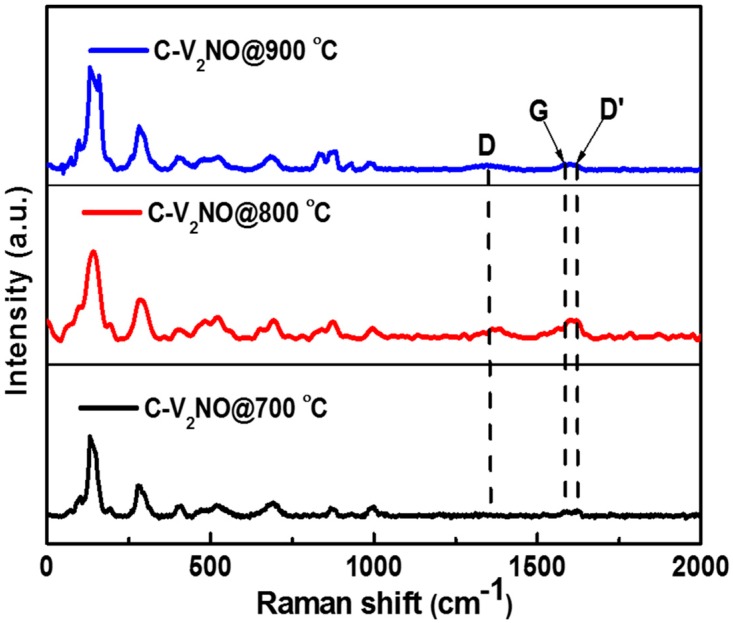
The Raman spectra of the C-V_2_NO materials grown at 700, 800 and 900 °C.

The existence of amorphous carbon in the nanomaterials was nonetheless further confirmed from the Raman spectra as shown in Figure 2 with the characteristic D, G and D’ bands. These three bands are attributed to carbonaceous material present in the C-V_2_NO nanostructures at different nitridation temperatures. The D peak position was observed at (1346–1373 cm^−1^) as indicated in Table 1 and is associated to the defects existing in the carbon lattice structure [28,29]. The G peak position observed at (1585–1593 cm^−1^) is related to the vibration modes of the graphitic sp^2^ carbon present [28,30]. A small shoulder was observed near the G peak suggesting a graphene-like carbon present in the C-V_2_NO nanomaterial. The D’ peak is ascribed to the lattice vibration mode of the G peak at the surface of the carbon structure [31,32]. A similar D’ peak has also been observed in boron carbon oxynitride films due to lattice distortions [33].

The high I_D_/I_G_ ratio of the C-V_2_NO@800 °C recorded in Table 1 suggests that the presence of defects could result from the nitrogen incorporated in the carbon lattice and also the strong interaction between the metal oxynitride and the carbon. The lower degree of graphitization (highest I_D_/I_G_ ratio) in the C-V_2_NO@800 °C can contribute to high electrochemical capacity [34,35]. The Raman spectra for the C-V_2_NO nanomaterials synthesized at 700, 800 and 900 °C also displayed Raman bands at wavenumbers of 142, 145, 192, 285, 479, 520.7, 693, 822, 873, and 995 cm^−1^ as seen in Figure 2 which can be attributed to the cubic symmetry vibration mode of the vanadium oxynitride [36] and the vanadium oxides (e.g., VO_2_ and V_2_O_5_) stretching mode [25].

**Figure 3:**
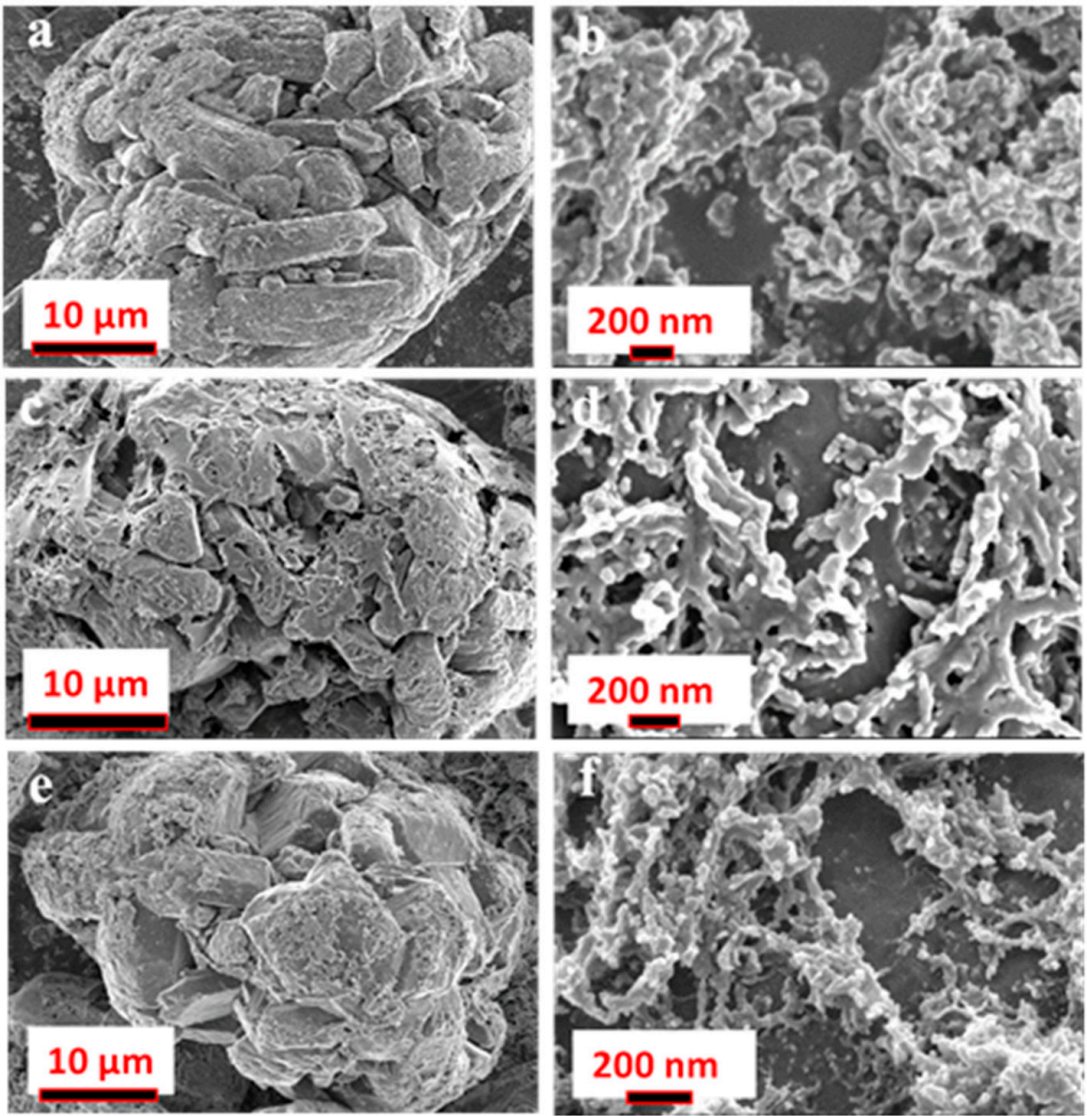
Scanning electron microscope (SEM) images at low and high magnification of the synthesized (**a**,**b**) C-V_2_NO@700 °C, (**c**,**d**) C-V_2_NO@800 °C and (**e**,**f**) C-V_2_NO@900 °C nanomaterials.

The morphological analysis of the C-V_2_NO nanomaterials synthesized at different temperatures is presented in Figure 3 at low and high magnification. The morphologies of the C-V_2_NO nanomaterials at different temperatures exhibited an agglomeration of cubic structures which showed a web-like morphology on the surface with irregular cavities (Figure 3a,c,e). At higher magnifications, C-V_2_NO@700 °C shows densely packed nanoparticles that are non-uniformly distributed (Figure 3b). In contrast, the surface morphology of the C-V_2_NO@800 °C shows interconnecting particles to form a web-like structure (Figure 3d). At 900 °C, a disintegrated network of the C-V_2_NO is observed (Figure 3f). In the C-V_2_NO@ 800 °C, it can be seen clearly that the porous cavities can penetrate deep into the material with a non-uniform distribution compared to the C-V_2_NO nanomaterials obtained at 700 °C and 900 °C.

**Figure 4:**
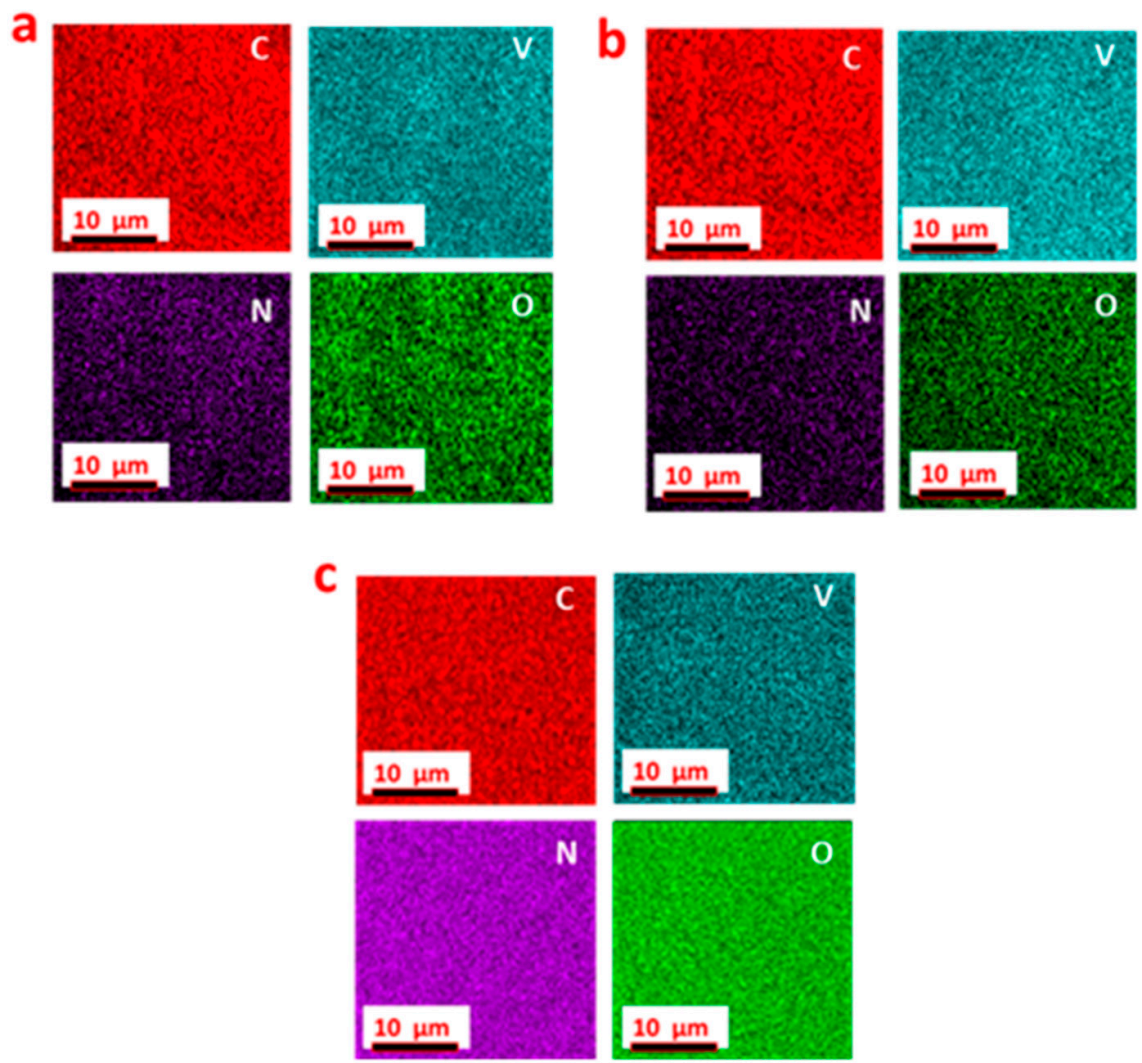
Distribution of individual elements in: (**a**) C-V_2_NO@700 °C, (**b**) C-V_2_NO@800 °C and (**c**) C-V_2_NO@900 °C materials.

In order to investigate the composition element at different nitridation, energy-dispersive X-ray spectroscopy (EDX) analysis was carried out to determine the elements present in the materials. The elemental mapping displayed the distinct element distribution in the C-V_2_NO@700 °C, C-V_2_NO@800 °C and C-V_2_NO@900 °C nanostructures as shown in Figure 4. This shows the presence of carbon (C), nitrogen (N), vanadium (V) and oxygen (O) atoms in all the C-V_2_NO nanomaterials. As seen in Figure 4a–c, a homogeneous distribution of the elements in the overall structure has been observed which confirms that the C-V_2_NO nanomaterials were successfully synthesized at different nitridation temperatures.

Table 2 shows the presence of carbon, nitrogen, vanadium and oxygen with their respective content. At 800 °C, the materials displayed higher C and N content as compared to the others. The oxygen content was found to decrease with the nitridation temperature. The different atomic composition values confirmed the dependence of the nitridation temperature on the element content which can affect the electrochemical performance of the materials.

**Figure 5:**
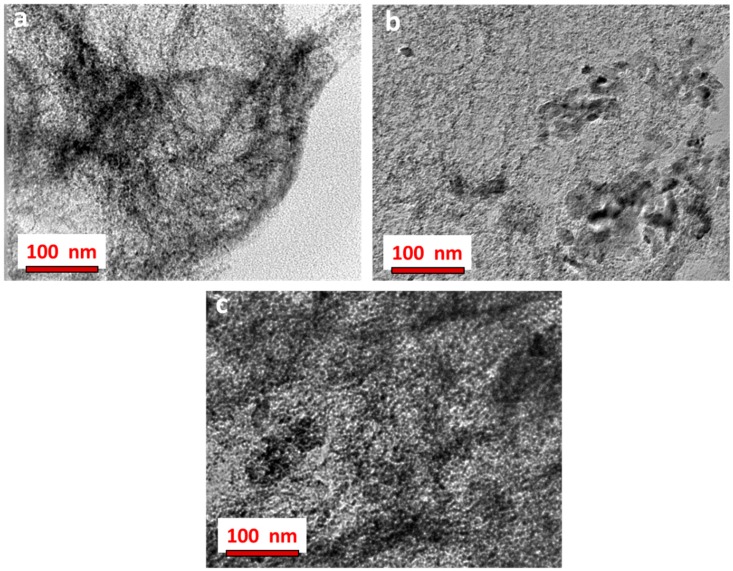
Transmission electron microscope (TEM) images of the synthesized C-V_2_NO nanomaterials: (**a**) C-V_2_NO@700 °C, (**b**) C-V_2_NO@800 °C and (**c**) C-V_2_NO@900 °C.

A more detailed analysis of the C-V_2_NO nanostructure was obtained from transmission electron microscopy (TEM) studies. Figure 5 shows the TEM images of all the nanomaterials which revealed that the materials could be viewed as interconnected porous web-like structures. Based on the XRD, Raman and SEM analyses, the selected area electron diffraction (SAED) pattern of the C-V_2_NO@800 °C nanomaterials was evaluated and presented in Figure S2 (Supporting information). It shows the electron diffraction planes at (111), (200), and (220) which corresponds to the vanadium oxynitride-carbon structure.

**Figure 6:**
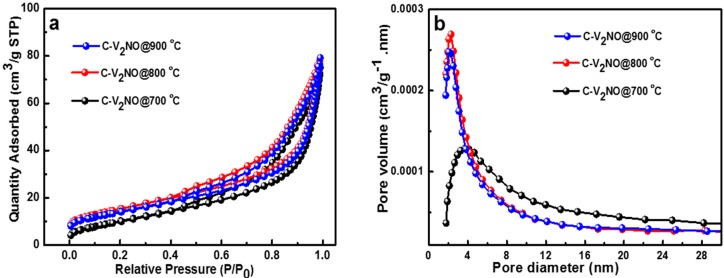
(**a**) N_2_ absorption/desorption isotherms and (**b**) pore size distribution of C-V_2_NO at 700–900 °C nitridation temperature.

To investigate the porosity properties of the nanomaterials, N_2_ adsorption-desorption measurement was carried out on C-V_2_NO nanomaterials at 700, 800 and 900 °C, as shown in Figure 6. The N_2_ isotherm of the C-V_2_NO nanomaterials in Figure 6a depicted a type IV adsorption–desorption isotherm with a H3 hysteresis behaviour associated with plate-like particles giving rise to slit-shaped pores [37]. The specific surface areas (SSAs) of the different carbon-vanadium oxynitride nanomaterials obtained were 91.6 ± 0.22 m^2^ g^−1^, 121.6 ± 0.83 m^2^ g^−1^ and 113 ± 0.74 m^2^ g^−1^ at 700, 800 and 900 °C, respectively. The synthesis of these mesoporous interconnected web-like structures with controlled growth temperature yielded higher value of the specific surface area as compared to those reported in the literature namely, 28.8 m^2^ g^−1^ for VO*_x_*N*_y_*, 40.8 m^2^ g^−1^ for VO*_x_*N*_y_*-C [22] and 60 m^2^ g^−1^ for TiO*_x_*N*_y_* [38], respectively.

Figure 6b shows the pore-size distribution with a pore diameter ranging from −2 to 8 nm which signifies the presence of a predominantly mesoporous C-V_2_NO material. It shows clearly that the pore diameter of the C-V_2_NO nanomaterial decrease with increasing nitridation temperature. The C-V_2_NO@700 °C shows a broad pore size distribution whereas a narrow pore size distribution was observed for the C-V_2_NO nanomaterials at 800 and 900 °C. As seen in Figure 6a,b, the C-V_2_NO@800 °C exhibited a higher specific surface area and a higher pore volume than the C-V_2_NO@700 °C and C-V_2_NO@900 °C which could be due to the highest ratio of disordered carbon relative to the graphitic carbon with a small pore diameter of 2 nm. The pore diameter value of the C-V_2_NO@800 °C (2 nm) is smaller than those reported for most reports on mesoporous oxynitride-based materials such as mesoporous bimetallic molybdenum tungsten oxynitride (4 nm) [39], zirconium doped perovskite oxynitride LaTaON_2_ (20 nm) [40], titanium oxynitride were grafted onto the surfaces of mesoporous silica (2.18 nm) [41]. This mesoporous structure of the C-V_2_NO@800 °C could greatly increase the mass transport of electrolytes ions within the electrodes for fast redox reactions and thus further enhance the electrochemical performance.

The formation of mesoporous carbon-vanadium oxynitride can be postulated to result from the reaction of vanadium metavanadate and melamine at higher temperatures. Typically, at high temperatures (>220 °C), the melamine decomposes to give –CH_2_, –C–N and nitrogen groups which form the matrix ring (N–C matrix) at higher temperatures [42] and the NH_4_VO_3_ decomposes at (>300 °C) to yield vanadium oxides, NH_3_ gas and H_2_O [43,44]. The NH_3_ gas can further reduce the vanadium oxides in the presence of –CH_2_ and N–C matrix from melamine decomposition leading to the formation of vanadium oxynitride material at higher temperatures (500–1500 °C) [45]. The presence of the metal oxide and N–C, as well as the NH_3_, favoured the formation of the carbon-vanadium oxynitride at high nitridation temperatures.

At 700 °C, small nanoparticles of C-V_2_NO were formed and connected in a web network with a moderate degree of graphitized carbon.

When the temperature was further increased to 800 °C, the particles are strongly interconnected to form a more mesoporous network (higher surface area) that can favour a better interaction of the C-V_2_NO surface with the electrolyte. Consequently, the carbon network became more defective resulting in a lower degree of graphitization. At 900 °C, however, the highly mesoporous network was disintegrated resulting in a decreased specific surface area and a moderately graphitized carbon network. Thus, nitridation temperature influenced the morphology, structure and surface area of the C-V_2_NO.

For all materials analysed, C-V_2_NO prepared at 800 °C portrayed good crystallinity, a higher ratio of disorder with a well-interconnected porous structure. More specifically, it exhibited a high specific surface area and yielded an efficient nanometer-sized pores (2 nm) and therefore, presented an optimal nitridation process that could be beneficial for enhanced electrochemical performance. Thus, the XPS analysis of the C-V_2_NO@800 °C was performed to determine the active species introduced into the material. Figure S3 (supporting information) shows the C, V, O and N peaks deconvoluted in high resolution into C_1s_ V_2p_, O_1s_ and N_1s_ which confirms the configurations of carbon, nitrogen, oxygen and all the different vanadium ions (V^2+^, V^3+^, V^4+^ and V^5+^) [10,25,46,47,48,49,50,51,52,53,54,55,56].

### Electrochemical Performance

**Figure 7:**
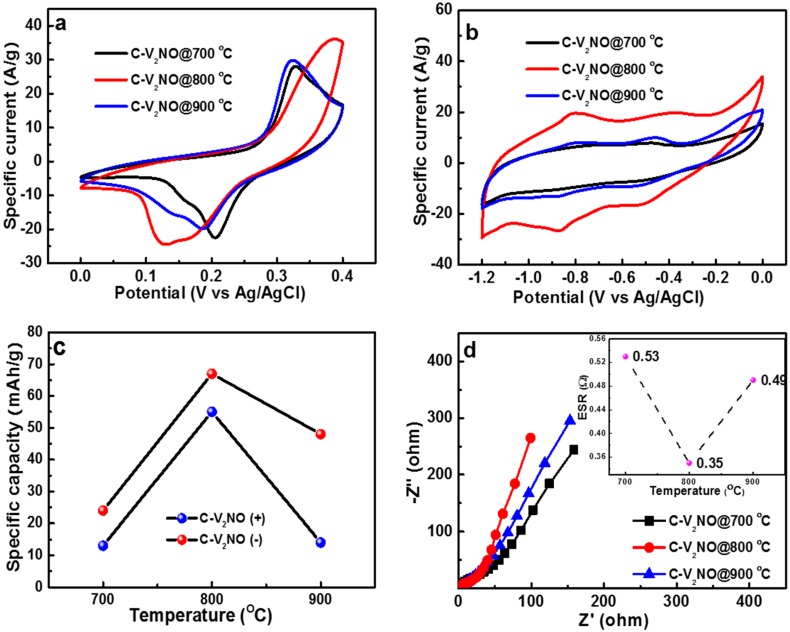
(**a**) The cyclic voltammetry (CV) curve of the C-V_2_NO electrodes in both positive (0 to 0.4 V), (**b**) negative (−1.2 to 0 V) potential window ranges at 50 mV s^−1^, (**c**) the specific capacity of the C-V_2_NO as positive and negative electrodes as a function of nitridation temperature at a specific current of 1 A g^−1^ and (**d**) the Nyquist plot (the inset is the equivalent series resistance (ESR) at different nitridation temperatures) for the C-V_2_NO electrodes material.

The electrochemical measurements of all the electrode materials were originally performed in a half-cell (three electrode) configuration using 6 M KOH aqueous electrolyte as seen in Figure 7. For comparison purposes, the electrochemical data of the nickel foam (NiF) as an electrode is given in the supplementary information (Figure S4). Figure 7a,b shows the cyclic voltammogram (CV) plots of the different C-V_2_NO nanomaterials in both positive (0 to 0.40 V) and negative (−1.20 to 0 V) potential window ranges at a 50 mV s^−1^ scan rate. As observed in these figures, the CV plots of C-V_2_NO@800 °C exhibits a higher current response compared to the other C-V_2_NO samples prepared at 700 and 900 °C in both operating potential ranges. All the CV curves of the electrodes present Faradaic behavior in the positive potential window and pseudocapacitive behavior in the negative potential window. As seen in Figure 7a, the CV curves of the nanomaterials show anodic and cathodic peaks shift. This kind of behaviour is normal for carbon-based materials measured in the positive potential widow using KOH electrolyte. The shift in the anodic peak observed from these three samples with 700 and 900 °C being different to that of 800 °C is due to carbon content and the amount of amorphous carbon that are close to each other for other temperatures as compared to 800 °C. Figure 7c shows clearly that the C-V_2_NO@800 °C electrode presents the highest specific capacity value as a function of the nitridation temperature in the positive and in the negative potential windows.

Figure 7d displays the Nyquist plot with the inset showing equivalent series resistance (ESR) as a function of nitridation temperature for different C-V_2_NO electrodes. More specifically, the C-V_2_NO@800 °C electrode shows smallest ESR, a shorter diffusion length and is closer to the vertical line (Z”-axis) than the other electrode materials. This low ESR value for 800 °C can be related to a good ion diffusion at the electrode–electrolyte interface and fast charge transport between the electrode and the current collector which are greatly influenced by the surface chemistry of the C-V_2_NO@800 °C electrode.

As can clearly be seen in the three-electrode configuration, the electrochemical measurements of the C-V_2_NO@800 °C depicted a superior electrochemical response based on the result in Figure 7. It also shows good stability in terms of capacity retention (94%) over 5000 cycles at 10 A g^−1^ as shown in the supplementary information (Figure S5). This is linked to the mesoporous web-like morphology of the C-V_2_NO@800 °C coupled with the high SSA and a small pore diameter which provides an efficient contact between the surface of electroactive web-like and the 6 M KOH even at high rates to build up a good path for fast electron transport [25]. In addition, the high I_D_/I_G_ ratio of the C-V_2_NO@800 °C yielded a good affinity for the 6 M KOH electrolyte ions due to the surface hydrophilicity and the good carbon surface wettability. From this result, the electrochemical measurements for a full-cell device is carried out using C-V_2_NO@800 °C samples as both negative and positive electrode to produce a symmetric device.

**Figure 8:**
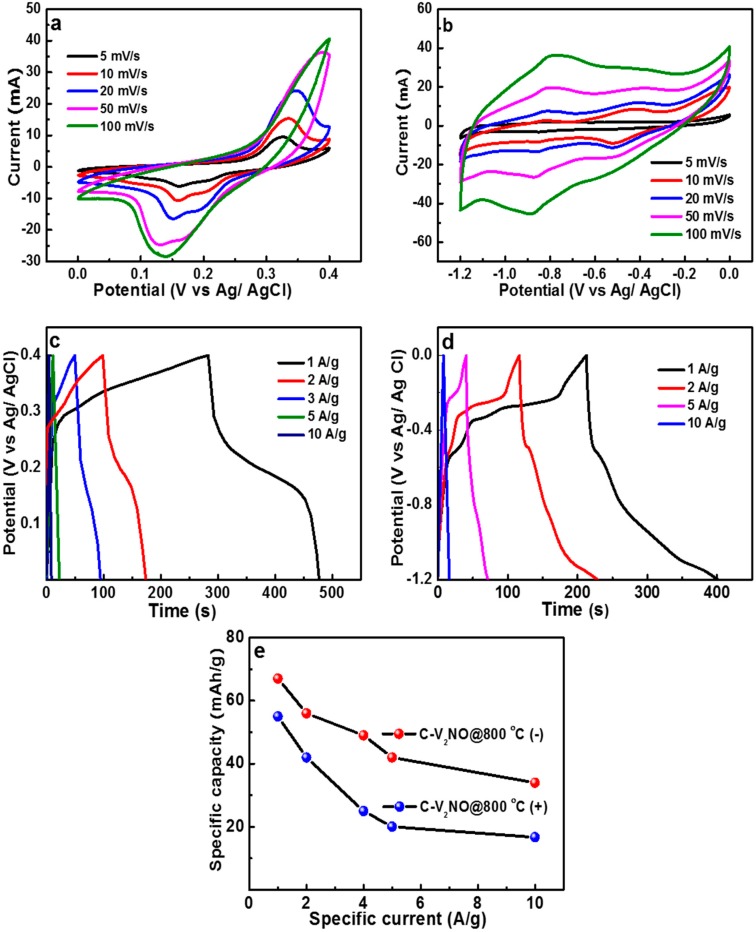
(**a**) CV plots at different scan rates, (**b**) galvanostatic charge-discharge (GCD) plots at different specific currents in a positive (0 to 0.4 V) potential window, (**c**) CV curves at different scan rates, (**d**) GCD curves at different specific currents in a negative (−1.2 to 0 V) potential window of and (**e**) specific capacity of both positive and negative electrodes against specific current.

Figure 8a,b shows the CV plot of the C-V_2_NO@800 °C electrode materials in both positive (0 to 0.4 V) and negative (−1.2 to 0 V) potential window ranges at different scan rates ranging from 5 to 100 mV s^−1^. The CV curve depicts a Faradaic electrode material in the positive potential window as seen in Figure 8a. This behaviour was confirmed by the presence of a pair of redox peaks at a scan rate of 50 mV s^−1^ which correspond to the anodic peak (−0.12 V) and cathodic peak (−0.35 V). Figure 8b shows a pseudocapacitive behavior in the negative potential window with two redox peaks during the anodic (−0.80, −0.38 V) and the cathodic (−0.87, −0.53 V).

These Faradaic and pseudocapacitive processes in the positive and negative potential windows are due to the presence of the vanadium oxide in the material which indicates a reversible redox reaction [57]. Based on the literature, vanadium oxides may lead to a redox reaction, but it is a challenge to assign the peak to an individual oxide [58].

Figure 8c displays the associated GCD plot at different specific current value. The existence of a Faradaic behaviour is depicted with the non-linear discharge profile in the potential window of 0 to 0.4 V. Figure 8d shows the GCD curves at different specific currents in the potential window of −1.2 to 0 V. It confirmed the existence of a pseudocapacitive behavior as shown in Figure 8b. Figure 8e shows the specific capacity of the C-V_2_NO@ 800 °C calculated in the positive and the negative potential window at different specific currents ranging from 1 to 10 A g^−1^. The specific capacities of the C-V_2_NO@800 °C were found to be 55 and 67 mA h g^−1^ in the positive and in the negative potential window, respectively, at a specific current of 1 A g^−1^.

**Figure 9:**
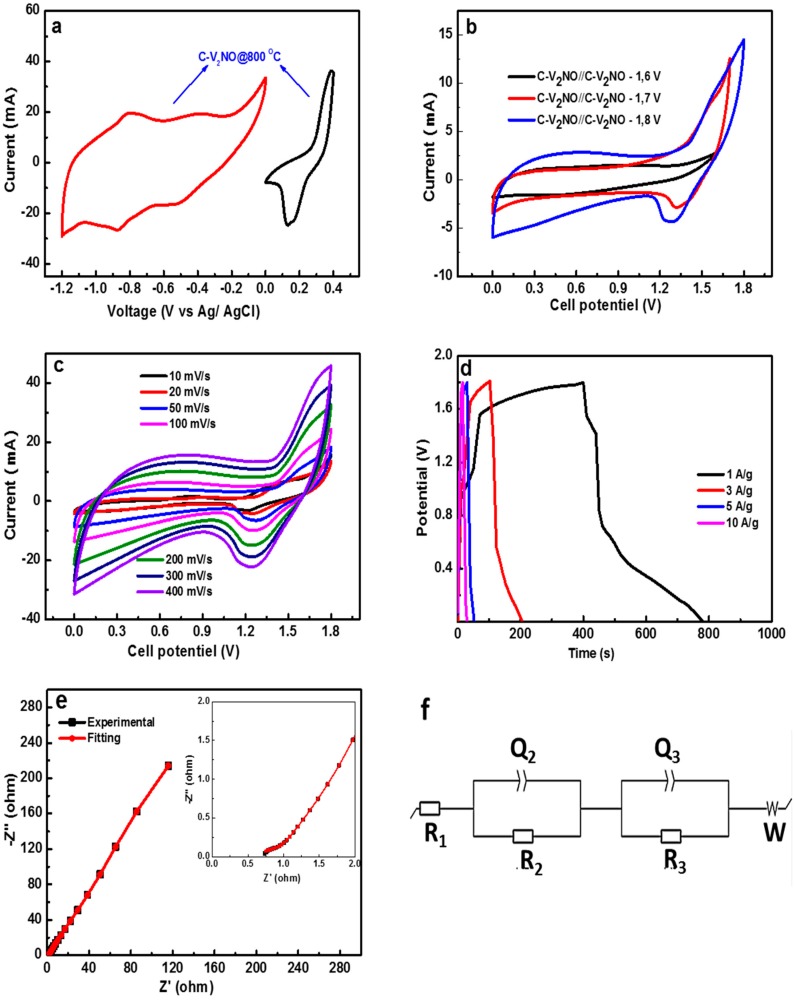
(**a**) Combined CV plots from the negative and positive potential window of the C-V_2_NO@800 °C electrode at 50 mV s^−1^, (**b**) CV at 1.6, 1.7 and 1.8 V at 30 mV s^−1^, (**c**) CV plot at different scan rates for cell potential of 1.8 V, (**d**) GCD plot of the device at different specific currents, (**e**) the fitted Nyquist plot with the electrochemical impedance spectroscopic (EIS) plot and (**f**) equivalent circuit of the C-V_2_NO//C-V_2_NO symmetric device.

To further demonstrate the electrochemical performance of the C-V_2_NO@800 °C electrode extensively, a symmetric cell was fabricated using the materials prepared at 800 °C as both positive and negative electrodes (C-V_2_NO//C-V_2_NO) in the same electrolyte (6 M KOH). Figure 9a shows the CV profiles of the C-V_2_NO@800 °C measured in stable working potential windows of −1.2 to 0 V and 0 to 0.4 V, respectively, at a scan rate of 50 mV s^−1^, and an estimation of the working potential window of 1.6 V could be expected from the symmetric device.

Figure 9b presents CV plots at different voltages such as 1.6, 1.7 and 1.8 V using a scan rate of 30 mV s^−1^. The cyclic voltammogram curve at 1.8 V shows the highest current response and good contribution of the two behaviours of C-V_2_NO@800 °C in both positive and negative voltages compared to other (1.6 and 1.7 V). Moreover, the current leap at large voltage of 1.8 V was not observed, implying the stability of the symmetric device in this voltage (Figure 9b). Therefore, it can be concluded that the maximum stable working potential of the C-V_2_NO//C-V_2_NO symmetric device is 1.8 V.

Figure 9c shows the CV curves of the C-V_2_NO//C-V_2_NO symmetric supercapacitor at different scan rates ranging from 5–400 mV s^−1^ in an operating voltage of 1.8 V. The CV curves indicate a combination and the contribution of pseudocapacitive and Faradaic charge storage mechanisms. As observed, the CV curves present the same behavior from 5 to 400 mV s^−1^ scan rates, indicating rapid redox reactions at high scan rates. The specific current increases proportionally as a function of increased scan rate indicating the good rate capability of the symmetric device. Figure 9d shows the GCD profiles of C-V_2_NO//C-V_2_NO symmetric device at different specific currents of 1–10 A g^−1^ with a large voltage of 0–1.8 V. The GCD curves exhibited faradaic behavior for the symmetric device in agreement with CV curves from Figure 9b. Figure 9e illustrates the Nyquist plot and the fitting data (seen in red solid-line) of the symmetric device. The ESR value obtained experimentally is 0.74 Ω which is close to the values recorded from the fitting data suggesting an excellent fit with the experimental Nyquist plot. Figure 9f shows the equivalent circuit adopted to fit the electrochemical impedance spectroscopic (EIS) using a software ZFIT/EC-Lab version 10.40. In the Figure, R_1_ is the equivalent series (solution) resistance, and R_2_ and Q_2_ represent the rate of resistance and the electrochemical Faradaic activity, respectively. The equivalent series resistance is associated with the ohmic resistance of the electrode. In the high frequency, the R_1_ in series with the constant phase element Q_2_ and is placed in parallel with the R_2_. In the low-frequency, a close vertical line has been shown which is associated with an ideal supercapacitor giving a mass capacitance, Q_3_. The Nyquist plot of the symmetric device presents a slight deviation from the *Y*-axis which is mainly associated with the leakage resistance R_3_ arising from the Faradaic charge transfer process. The Q_3_ is in parallel to the R_3_ which is in series with the Warburg impedance characteristic element (W) [59,60].

**Figure 10:**
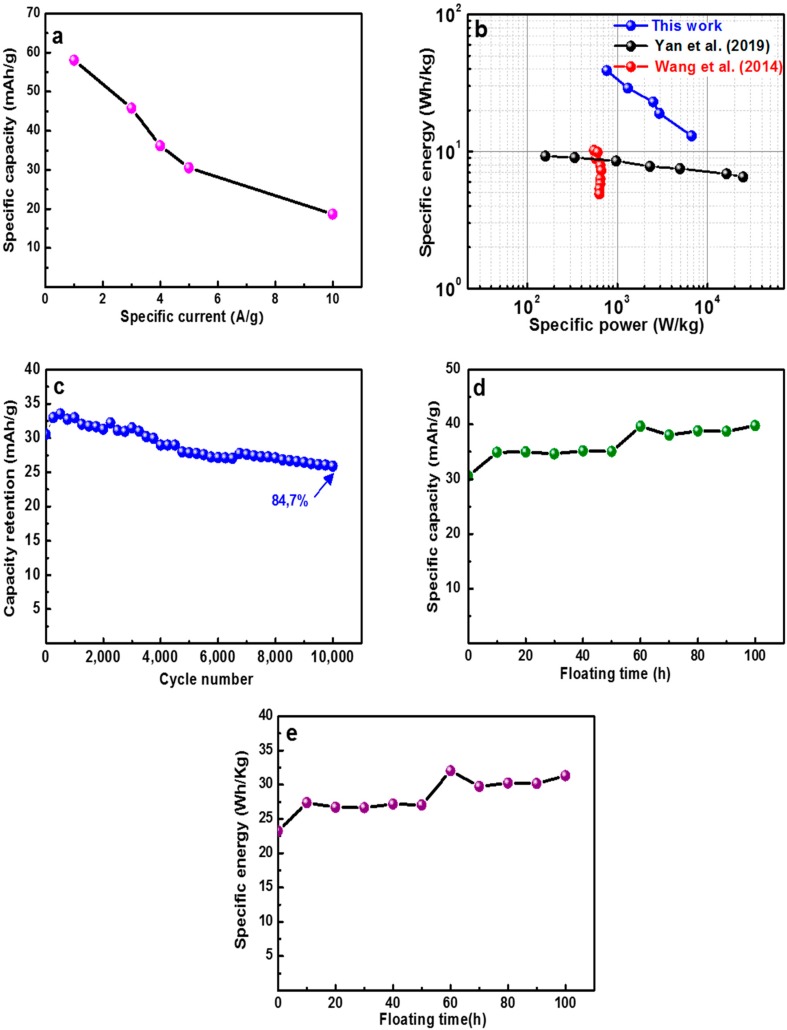
(**a**) Specific capacity of the C-V_2_NO//C-V_2_NO at different specific current in the full-cell configuration, (**b**) Ragone plot, (**c**) stability test for up to 10,000 cycles, (**d**) specific capacity and (**e**) energy density against a floating time of 100 h at 10 A g^−1^.

Figure 10a presents the profile of the specific capacity as a function of specific current. A specific capacity value of 58 mA h g^−1^ of the symmetric device was recorded at 1 A g^−1^. This was retained at 18.7 mA h g^−1^ for a specific current of 10 A g^−1^. Figure 10b exhibits the Ragone plot which describes the power density against energy density for the symmetric capacitor using different specific currents range of 1–10 A g^−1^. The specific energy of the C-V_2_NO//C-V_2_NO was 38 W h kg^−1^ with a related specific power of 764 W kg^−1^ at a specific current of 1 A g^−1^. At 10 A g^−1^ the specific energy was 12.4 W h kg^−1^ with an equivalent specific power of 6.6 kW kg^−1^. These values are superior to those reported for other symmetric oxy/nitride-based materials as shown in Figure 10b. For instance, Wang et al. [61] successfully synthesized MnO_2_/TiO_1−*x*_N*_x_* nanomaterials by depositing MnO_2_ on TiO_0.54_N_0.46_ nanotubes. The TiO_1−*x*_N*_x_* was prepared by using a CVD (chemical vapor deposition) system in an ammonia atmosphere at high temperature. The symmetric configuration delivered an specific energy value of 9.8 W h kg^−1^ and a specific power value of 620 W kg^−1^ at 100 A g^−1^ using 1 M KCl electrolyte [61]. Recently, Yan et al. [62] prepared a carbon anchored titanium carbonitride materials using a semi-solid-state carbonitridation route. The electrochemical performance of the symmetric cell can store a specific energy of 8.6 W h kg^−1^ and deliver a specific power of 160.5 W kg^−1^ at a specific current of 1 A g^−1^ in PVA/H_2_SO_4_ electrolyte [62].

In this work, the high specific energy and power value of the C-V_2_NO//C-V_2_NO cell observed can be attributed to the excellent electrochemical performance of the C-V_2_NO@800 °C working in a stable voltage of 1.8 V.

To evaluate the device stability of the C-V_2_NO//C-V_2_NO, constant cycling (charging–discharging) and floating time (voltage holding) tests were performed to study device degradation with varying parameters. Figure 10c shows the plot of capacity retention of the symmetric device as a function of cycle number at a high gravimetric current of 10 A g^−1^.

The cycling stability presented a good capacity retention of 84.7% up to 10,000 cycles, suggesting a good electrochemical stability of the symmetric cell. The floating test was evaluated after 10,000 stability test at 10 A g^−1^ by monitoring the specific capacity after every 10 h interval for up to 100 h and the result is shown in Figure 10d. The specific capacity value considerably increased after 50 h of voltage holding. This appreciable improvement can be seen clearly after calculating the specific energy for each voltage holding time as shown in Figure 10e. Interestingly, the specific energy value after 50 h improved from 23 to 27 W h kg^−1^ before reaching a plateau at a value of 32 W h kg^−1^, corresponding to an increase of 27% from the original value of 23 W h kg^−1^. This increase in specific capacity and specific energy can be associated to the increase in accessibility of the ions to initially inaccessible redox sites which could increase the wettability and a large diffusion of ions between the electrolyte/electrode during the long floating time (100 h) [63]. The cycling stability and the floating test of the device indicated that the symmetric device showed a good long-term stability without any significant degradation.

## 4. Conclusions

In this study, a porous carbon-vanadium oxynitride (C-V_2_NO) nanostructure was obtained at different nitridation temperatures of 700, 800 and 900 °C using a thermal decomposition method. All the synthesized nanomaterials exhibited single phase C-V_2_NO cubic structures. The BET analysis of the C-V_2_NO nanomaterials showed a good specific surface area (SSA) (from 91.6 to 121.6 m^2^ g^−1^) with a pore diameter ranging from −2 to 8 nm. The nitridation temperature played a crucial role in modulating the C-V_2_NO crystallinity, surface area, and elemental composition. The C-V_2_NO@700 °C nanostructure displayed a low surface area and a moderate degree of graphitization. The C-V_2_NO@800 °C nanomaterials showed a higher ratio of disorder with a well interconnected cavities structure and a narrower pore size distribution. In contrast, C-V_2_NO@900 °C gave a disintegrated network with a moderate degree of graphitization and a decrease in surface area compared to the C-V_2_NO@800 °C. Therefore, a nitridation temperature of 800 °C was found to be the optimal temperature for the generation of a suitable C-V_2_NO nanostructure with the highest electrochemical performance in both the positive and negative potential windows. A symmetric capacitor was fabricated using C-V_2_NO@800 °C revealing a specific energy value of 38 W h kg^−1^ and a specific power value of 764 W kg^−1^ at 1 A g^−1^ in a voltage of 1.8 V. The C-V_2_NO//C-V_2_NO exhibited a good cycling stability with 84.7% capacity retention up to 10,000 cycles at 10 A g^−1^. This excellent stability performance of the device was also confirmed via floating/aging test for up to 100 h showing a further improved device electrochemical performance.

## Figures and Tables

**Table 1:** D, G, D’ band positions and I_D_/I_G_ ratio of the C-V_2_NO samples.

Material	D Peak Position (cm^−1^)	G Peak Position (cm^−1^)	D’ Peak Position (cm^−1^)	I_D_/I_G_ Ratio (±0.03)
C-V_2_NO@700 °C	1349	1585	1611	0.47
C-V_2_NO@800 °C	1373	1593	1621	0.84
C-V_2_NO@900 °C	1346	1588	1618	0.56

**Table 2:** Carbon, vanadium, nitrogen and oxygen contents in the C-V_2_NO materials.

Material	C (Wt%)	V (Wt%)	N (Wt%)	O (Wt%)	Total
C-V_2_NO@700 °C	62	17	5	16	100
C-V_2_NO@800 °C	66	11	9	14	100
C-V_2_NO@900 °C	64	18	7	11	100

## Supplementary Materials

The following are available online at https://www.mdpi.com/2079-4991/9/12/1762/s1, Figure S1: The TEM images of the C-V_2_NO@800 °C nanomaterials, Figure S2: The selected area electron diffraction (SAED) pattern of the C-V_2_NO@800 °C nanomaterials, Figure S3: (a) Wide scan XPS spectrum of the C-V_2_NO@800 °C materials, indicating deconvoluted spectra of (b) V_2p_ binding energy region, (c) C_1s_ binding energy region, (d) N1s binding energy region and (e) O_1s_ binding energy regions, respectively, Figure S4: CV plot of the Ni foam and active material at a scan rate of 20 mV s^−1^, Figure S5: Coulombic efficiency and capacity retention as a function of cycle number at a specific current of 10 A g^−1^ C-V_2_NO@800 °C.

## References

[1] Surendran S., Shanmugapriya S., Shanmugam S., Vasylechko L., Kalai Selvan R. (2018). Interweaved Nickel Phosphide Sponge as an Electrode for Flexible Supercapattery and Water Splitting Applications. ACS Appl. Energy Mater..

[2] Huang P., Lethien C., Pinaud S., Brousse K., Laloo R., Turq V., Respaud M., Demortiere A., Daffos B., Taberna P.L. (2016). On-chip and freestanding elastic carbon films for micro-supercapacitors. Science.

[3] Kang Y.J., Chung H., Han C.-H., Kim W. (2012). All-solid-state flexible supercapacitors based on papers coated with carbon nanotubes and ionic-liquid-based gel electrolytes. Nanotechnology.

[4] Su F., Miao M. (2014). Asymmetric carbon nanotube–MnO2 two-ply yarn supercapacitors for wearable electronics. Nanotechnology.

[5] Deng L., Zhang G., Kang L., Lei Z., Liu C., Liu Z.-H.H. (2013). Graphene/VO2 hybrid material for high performance electrochemical capacitor. Electrochim. Acta.

[6] Simon P., Gogotsi Y. (2008). Materials for electrochemical capacitors. Nat. Mater..

[7] Yu L., Chen G.Z. (2016). High energy supercapattery with an ionic liquid solution of LiClO _4_. Faraday Discuss..

[8] Yu L., Chen G.Z. (2016). Redox electrode materials for supercapatteries. J. Power Sour..

[9] Zhi M., Xiang C., Li J., Li M., Wu N. (2013). Nanostructured carbon–metal oxide composite electrodes for supercapacitors: a review. Nanoscale.

[10] Silversmit G., Depla D., Poelman H., Marin G.B., De Gryse R. (2004). Determination of the V2p XPS binding energies for different vanadium oxidation states (V5+ to V0+). J. Electron Spectrosc. Relat. Phenom..

[11] Cherusseri J., Sambath Kumar K., Choudhary N., Nagaiah N., Jung Y., Roy T., Thomas J. (2019). Novel mesoporous electrode materials for symmetric, asymmetric and hybrid supercapacitors. Nanotechnology.

[12] Yu J., Gao X., Cui Z., Jiao Y., Zhang Q., Dong H., Yu L., Dong L. (2019). Facile Synthesis of Binary Transition Metal Sulfide Tubes Derived from NiCo-MOF-74 for High-Performance Supercapacitors. Energy Technol..

[13] He T., Wang Z., Li X., Tan Y., Liu Y., Kong L., Kang L., Chen C., Ran F. (2019). Intercalation structure of vanadium nitride nanoparticles growing on graphene surface toward high negative active material for supercapacitor utilization. J. Alloys Compd..

[14] Kumar U.N., Ghosh S., Thomas T. (2019). Metal Oxynitrides as Promising Electrode Materials for Supercapacitor Applications. ChemElectroChem.

[15] Zheng S., Gao L. (2006). Nanosized Titanium Oxynitride and Molybdenum Oxynitride Particles Assembled in Mesoporous Silica MCM-41. Mater. Sci. Forum.

[16] Zhao Y.M., Hu W.B., Xia Y.D., Smith E.F., Zhu Y.Q., Dunnill C.W., Gregory D.H. (2007). Preparation and characterization of tungsten oxynitride nanowires. J. Mater. Chem..

[17] Zheng Q., Kvit A., Cai Z., Ma Z., Gong S. (2017). A freestanding cellulose nanofibril–reduced graphene oxide–molybdenum oxynitride aerogel film electrode for all-solid-state supercapacitors with ultrahigh energy density. J. Mater. Chem. A.

[18] Porto R.L., Frappier R., Ducros J.B.B., Aucher C., Mosqueda H., Chenu S., Chavillon B., Tessier F., Cheviré F., Brousse T. (2012). Titanium and vanadium oxynitride powders as pseudo-capacitive materials for electrochemical capacitors. Electrochim. Acta.

[19] Ghosh S., Jeong S.M., Polaki S.R. (2018). A review on metal nitrides/oxynitrides as an emerging supercapacitor electrode beyond oxide. Korean J. Chem. Eng..

[20] Cheng F., He C., Shua D., Chen H., Zhang J., Tang S., Finlow D.E. (2011). Preparation of nanocrystalline VN by the melamine reduction of V2O5 xerogel and its supercapacitive behavior. Mater. Chem. Phys..

[21] Choi D., Blomgren G.E., Kumta P.N. (2006). Fast and Reversible Surface Redox Reaction in Nanocrystalline Vanadium Nitride Supercapacitors. Adv. Mater..

[22] Shu D., Cheng H., Lv C., Asi M.A., Long L., He C., Zou X., Kang Z. (2014). Soft-template synthesis of vanadium oxynitride-carbon nanomaterials for supercapacitors. Int. J. Hydrog. Energy.

[23] Zhou X., Shang C., Gu L., Dong S., Chen X., Han P., Li L., Yao J., Liu Z., Xu H. (2011). Mesoporous Coaxial Titanium Nitride-Vanadium Nitride Fibers of CoreÀshell Structures for High-Performance Supercapacitors. ACS Appl. Mater. Interfaces.

[24] Jacke S., Song J., Dimesso L., Brötz J., Becker D., Jaegermann W. (2011). Temperature dependent phosphorous oxynitride growth for all-solid-state batteries. J. Power Sour..

[25] Ndiaye N.M., Sylla N.F., Ngom B.D., Barzegar F., Momodu D., Manyala N. (2019). High-performance asymmetric supercapacitor based on vanadium dioxide/activated expanded graphite composite and carbon-vanadium oxynitride nanostructures. Electrochim. Acta.

[26] Ndiaye N.M., Madito M.J., Ngom B.D., Masikhwa T.M., Mirghni A.A., Manyala N. (2019). High-performance asymmetric supercapacitor based on vanadium dioxide and carbonized iron-polyaniline electrodes. AIP Adv..

[27] Ndiaye N.M., Ngom B.D., Sylla N.F., Masikhwa T.M., Madito M.J., Momodu D., Ntsoane T., Manyala N. (2018). Three dimensional vanadium pentoxide/graphene foam composite as positive electrode for high performance asymmetric electrochemical supercapacitor. J. Colloid Interface Sci..

[28] Chhowalla M., Ferrari A.C., Robertson J., Amaratunga G.A.J. (2000). Evolution of sp2 bonding with deposition temperature in tetrahedral amorphous carbon studied by Raman spectroscopy. Appl. Phys. Lett..

[29] Roy D., Chhowalla M., Wang H., Sano N., Alexandrou I., Clyne T.W., Amaratunga G.A.J. (2003). Characterisation of carbon nano-onions using Raman spectroscopy. Chem. Phys. Lett..

[30] Mohiuddin T.M.G., Lombardo A., Nair R.R., Bonetti A., Savini G., Jalil R., Bonini N., Basko D.M., Galiotis C., Marzari N. (2009). Uniaxial strain in graphene by Raman spectroscopy: G peak splitting, Grüneisen parameters, and sample orientation. Phys. Rev. B Condens. Matter Mater. Phys..

[31] Barzegar F., Bello A., Momodu D., Madito M.J., Dangbegnon J., Manyala N. (2016). Preparation and characterization of porous carbon from expanded graphite for high energy density supercapacitor in aqueous electrolyte. J. Power Sour..

[32] Fabiane M., Madito M.J., Bello A., Manyala N. (2017). Raman spectroscopy and imaging of Bernal-stacked bilayer graphene synthesized on copper foil by chemical vapour deposition: Growth dependence on temperature. J. Raman Spectrosc..

[33] Matsoso B.J., Ranganathan K., Mutuma B.K., Lerotholi T., Jones G., Coville N.J. (2017). Synthesis and characterization of boron carbon oxynitride films with tunable composition using methane, boric acid and ammonia. New J. Chem..

[34] Nikiel L., Jagodzinski P.W. (1993). Raman spectroscopic characterization of graphites: A re-evaluation of spectra/structure correlation. Carbon.

[35] Yang S., Song X., Zhang P., Gao L. (2015). Heating-Rate-Induced Porous α-Fe2O3 with Controllable Pore Size and Crystallinity Grown on Graphene for Supercapacitors. ACS Appl. Mater. Interfaces.

[36] Kafizas A., Hyett G., Parkin I.P. (2009). Combinatorial atmospheric pressure chemical vapour deposition (cAPCVD) of a mixed vanadium oxide and vanadium oxynitride thin film. J. Mater. Chem..

[37] Yu J., Wang G., Cheng B., Zhou M. (2007). Effects of hydrothermal temperature and time on the photocatalytic activity and microstructures of bimodal mesoporous TiO2 powders. Appl. Catal. B Environ..

[38] Wang Z., Li Z., Zou Z. (2015). Application of binder-free TiOxN1−x nanogrid film as a high-power supercapacitor electrode. J. Power Sour..

[39] Kartachova O., Glushenkov A.M., Chen Y., Zhang H., Chen Y. (2013). Bimetallic molybdenum tungsten oxynitride: Structure and electrochemical properties. J. Mater. Chem. A.

[40] Wang Y., Jin S., Pan G., Li Z., Chen L., Liu G., Xu X. (2019). Zr doped mesoporous LaTaON_2_ for efficient photocatalytic water splitting. J. Mater. Chem. A.

[41] Nada A.A., Bekheet M.F., Roualdes S., Gurlo A., Ayral A. (2019). Functionalization of MCM-41 with titanium oxynitride deposited via PECVD for enhanced removal of methylene blue. J. Mol. Liq..

[42] Levchik S.V., Balabanovich A.I., Levchik G.F., Costa L. (1997). Effect of melamine and its salts on combustion and thermal decomposition of polyamide 6. Fire Mater..

[43] Twu J., Shih C.-F., Guoa T.-H., Chenb K.-H. (1997). Raman spectroscopic studies of the thermal decomposition mechanism of ammonium metavanadate. J. Mater. Chem..

[44] Selim S.A., Philip C.A., Mikhail R.S. (1980). Thermal decomposition of ammonium metavanadate. Thermochim. Acta.

[45] Brown M.E., Glasser L., Stewart B.V. (1974). The thermal decomposition of ammonium metavanadate II The kinetics and mechanism of the decomposition. J. Therm. Anal..

[46] Gao B., Li X., Guo X., Zhang X., Peng X., Wang L., Fu J., Chu P.K., Huo K. (2015). Nitrogen-Doped Carbon Encapsulated Mesoporous Vanadium Nitride Nanowires as Self-Supported Electrodes for Flexible All-Solid-State Supercapacitors. Adv. Mater. Interfaces.

[47] Dwivedi N., Kumar S., Carey J.D., Malik H.K. (2012). Govind Photoconductivity and characterization of nitrogen incorporated hydrogenated amorphous carbon thin films. J. Appl. Phys..

[48] Liu Y., Liu L., Tan Y., Kong L., Kang L., Ran F. (2018). Well-Dispersed Vanadium Nitride on Porous Carbon Networks Derived from Block Copolymer of PAN-b-PDMC-b-PAN Absorbed with Ammonium Metavanadate for Energy Storage Application. J. Phys. Chem. C.

[49] Hryha E., Rutqvist E., Nyborg L. (2012). Stoichiometric vanadium oxides studied by XPS. Surf. Interface Anal..

[50] Ureña-Begara F., Crunteanu A., Raskin J.P. (2017). Raman and XPS characterization of vanadium oxide thin films with temperature. Appl. Surface Sci..

[51] Marton D., Boyd K.J., Al-Bayati A.H., Todorov S.S., Rabalais J.W. (1994). Carbon nitride deposited using energetic species: A two-phase system. Phys. Rev. Lett..

[52] Sevilla M., Fuertes A.B. (2009). The production of carbon materials by hydrothermal carbonization of cellulose. Carbon.

[53] Rantho M.N., Madito M.J., Manyala N. (2018). Symmetric supercapacitor with supercapattery behavior based on carbonized iron cations adsorbed onto polyaniline. Electrochim. Acta.

[54] Mutuma B.K., Matsoso B.J., Ranganathan K., Keartland J.M., Wamwangi D., Coville N.J. (2017). Generation of radical species in CVD grown pristine and N-doped solid carbon spheres using H _2_ and Ar as carrier gases. RSC Adv..

[55] Hong Z., Shen B., Chen Y., Lin B., Gao B. (2013). Enhancement of photocatalytic H2 evolution over nitrogen-deficient graphitic carbon nitride. J. Mater. Chem. A.

[56] Fang J., Fan H., Li M., Long C. (2015). Nitrogen self-doped graphitic carbon nitride as efficient visible light photocatalyst for hydrogen evolution. J. Mater. Chem. A.

[57] Deng Y., Xie Y., Zou K., Ji X. (2016). Review on recent advances in nitrogen-doped carbons: preparations and applications in supercapacitors. J. Mater. Chem. A.

[58] Ghimbeu C.M., Raymundo-Piñero E., Fioux P., Béguin F.F., Vix-Guterl C. (2011). Vanadium nitride/carbon nanotube nanocomposites as electrodes for supercapacitors. J. Mater. Chem..

[59] Barzegar F., Bello A., Dangbegnon J.K., Manyala N., Xia X. (2017). Asymmetric supercapacitor based on activated expanded graphite and pinecone tree activated carbon with excellent stability. Appl. Energy.

[60] Mirghni A.A., Oyedotun K.O., Mahmoud B.A., Bello A., Ray S.C., Manyala N. (2019). Nickel-cobalt phosphate/graphene foam as enhanced electrode for hybrid supercapacitor. Compos. Part B Eng..

[61] Wang Z., Li Z., Feng J., Yan S., Luo W., Liu J., Yu T., Zou Z. (2014). MnO2 nanolayers on highly conductive TiO0.54N0.46 nanotubes for supercapacitor electrodes with high power density and cyclic stability. Phys. Chem. Chem. Phys..

[62] Yan H., Wang J., Fang Y., Zhou M., Guo X., Wang H.-Q., Dai Y., Li W., Zheng J.-C. (2019). Porous carbon anchored titanium carbonitride for high-performance supercapacitor. Electrochim. Acta.

[63] Sylla N.F., Ndiaye N.M., Ngom B.D., Momodu D., Madito M.J., Mutuma B.K., Manyala N. (2019). Efect of porosity enhancing agents on the electrochemical performance of high-energy ultracapacitor electrodes derived from peanut shell waste. Sci. Rep..

