# Supplementary material for: Nitridation Temperature Effect on Carbon Vanadium Oxynitrides for a Symmetric Supercapacitor
Source: Nanomaterials (Basel). 2019 Dec 11;9(12):1762. doi: 10.3390/nano9121762 (PMC6956286; doi:10.3390/nano9121762)
Supplement: Supplementary file 1 [file nanomaterials-09-01762-s001.pdf]

Supplementary Materials: Nitridation Temperature Effect on Carbon Vanadium Oxynitrides for a Symmetric Supercapacitor

Ndeye M. Ndiaye [1], Ndeye F. Sylla [1], Balla D. Ngom [2], Bridget K. Mutuma [1], Julien K. Dangbegnon [1], Sekhar C. Ray [3] and Ncholu Manyala [1,*]

[1] Department of Physics, Institute of Applied Materials, SARChI Chair in Carbon Technology and Materials, University of Pretoria, Pretoria 0028, South Africa; nmaty.ndiaye@gmail.com (N.M.N.); ntoufasylla@gmail.com (N.F.S); bridgetmutuma@gmail.com (B.K.M.); dangbegnon01@googlemail.com (J.K.D.)

[2] Laboratoire de Photonique Quantique d'Energie et de NanoFabrication, Groupe de Physique du Solide et Science des Materiaux, Departement de Physique FST-UCAD BP 5005 Dakar-Fan, Dakar 999066, Senegal; bdngom@gmail.com

[3] Department of Physics, College of Science, Engineering and Technology, University of South Africa, Private Bag X6, Florida 1710, Science Campus, Christiaan de Wet and Pioneer Avenue, Florida Park, Johannesburg 1710, South Africa; raysc@unisa.ac.za

* Correspondence: ncholu.manyala@up.ac.za; Tel.: +27-12-420-3549; Fax.: +27-12-420-2516

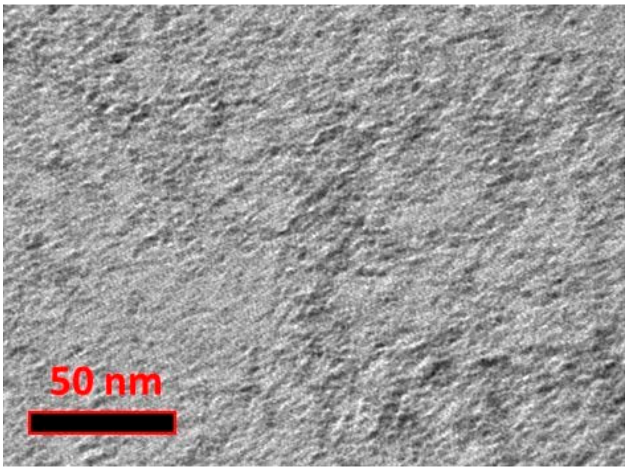

Figure S1: The TEM images of the C-V_2NO@800 °C nanomaterials

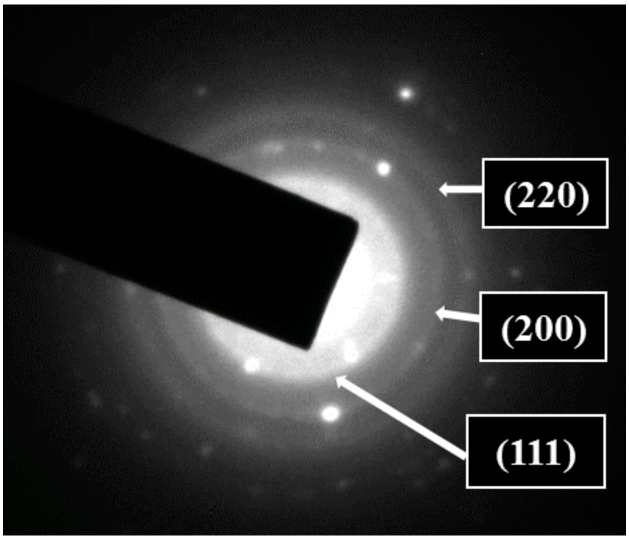

Figure S2: The selected area electron diffraction (SAED) pattern of the C-V_2NO@800 °C nanomaterials

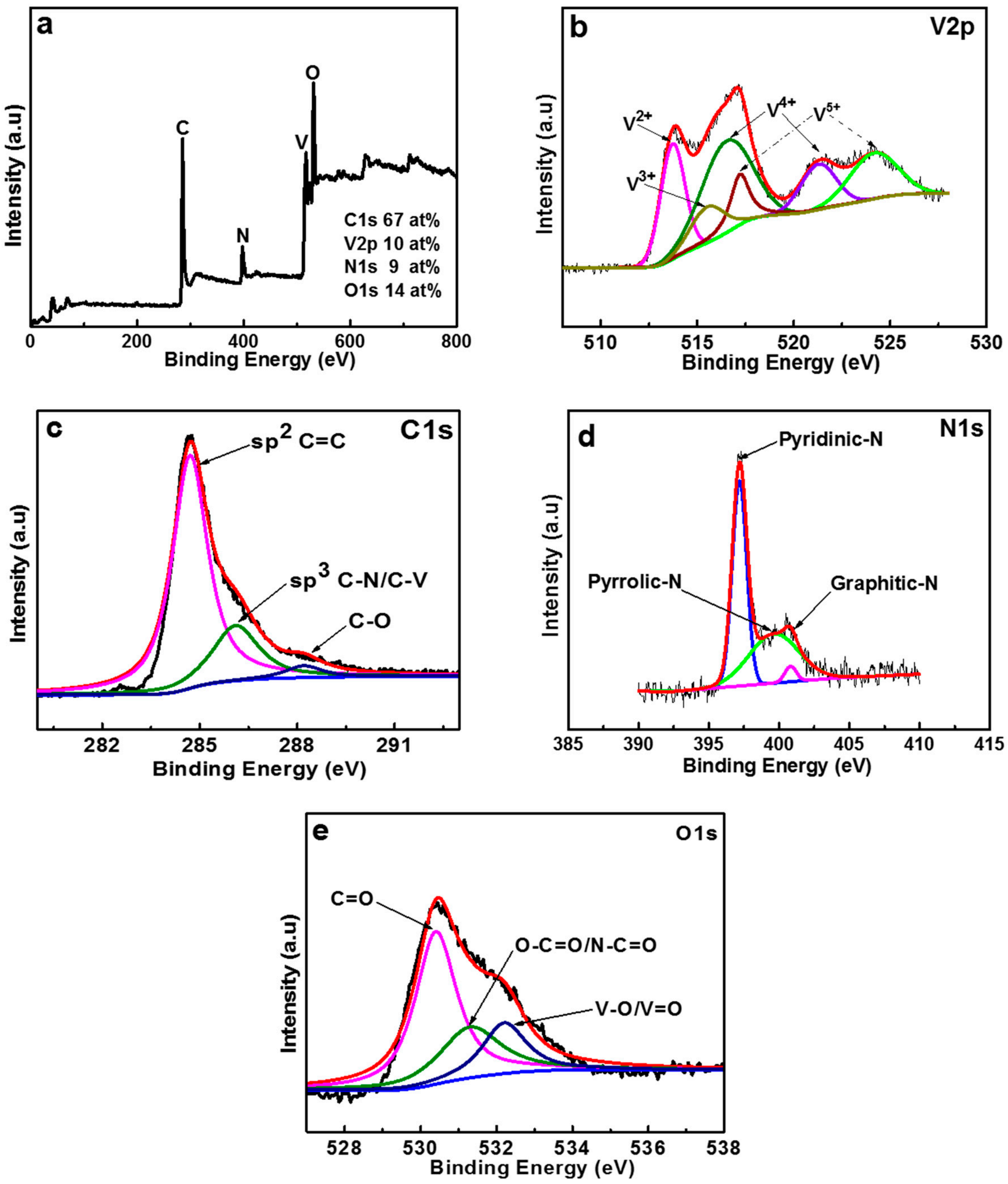

Figure S3: (**a**) Wide scan XPS spectrum of the C-V_2NO@800 °C materials, indicating deconvoluted spectra of (**b**) V2p binding energy region, (**c**) C1s binding energy region, (**d**) N1s binding energy region and (**e**) O1s binding energy regions, respectively.

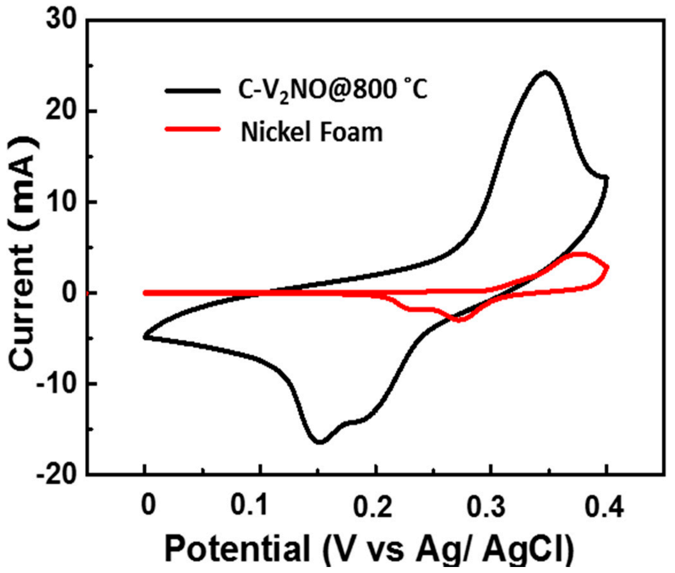

Figure S4: CV plot of the Ni foam and active material at a scan rate of 20 mV s^{-1}

Figure S3 shows the CV curves of nickel foam (NiF) and active material (C-V_2NO@800 °C) at a scan rate of 20 mV s^{-1} in a potential window range of 0–0.4 V. As clearly observed, the CV curve of the active materials presents a superior current response as compared to the CV plot of the current collector. This high current response of the C-V_2NO@800 °C confirms that the current collector (NiF) does not show an effect on the electrochemical performance of the C-V_2NO@800 °C nanomaterials.

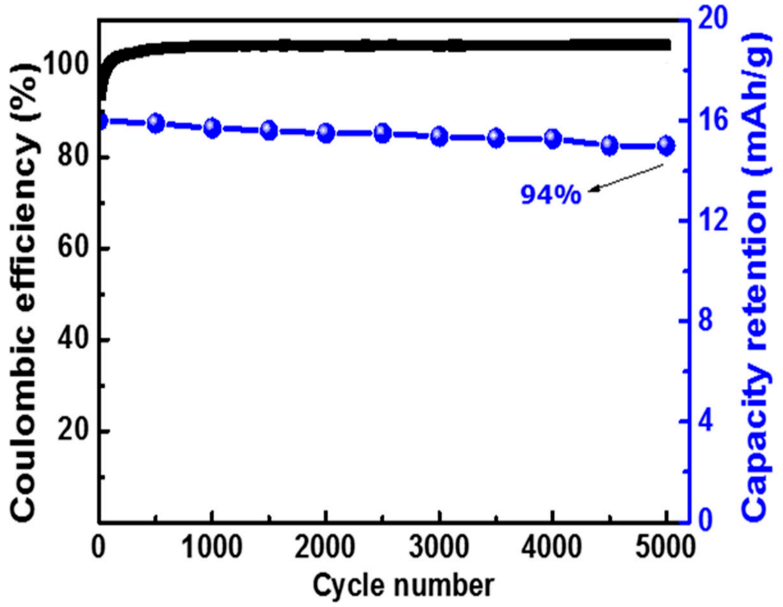

Figure S5: Coulombic efficiency and capacity retention as a function of cycle number at a specific current of 10 A g^{-1} C-V_2NO@800 °C
